# Member commitment in farmers’ cooperatives in China: The role of contractual and relational governance mechanisms

**DOI:** 10.1371/journal.pone.0288925

**Published:** 2023-07-27

**Authors:** Lijun Zeng, Junyi Wan, Qinying He

**Affiliations:** 1 School of Finance and Economics, Guangdong Polytechnic Normal University, Guangzhou, China; 2 College of Economics and Management, South China Agricultural University, Guangzhou, China; Wroclaw University of Environmental and Life Sciences: Uniwersytet Przyrodniczy we Wroclawiu, POLAND

## Abstract

Farmers’ cooperatives play a critical role in social, economic, and environmental sustainability in terms of poverty reduction, food quality and safety, farm sustainability, and members’ well-being. However, they are generally faced with low or declining member commitment, which restricts their performance and sustainable development. This study aims to investigate the effect of cooperative governance on member commitment as well as the moderating effects of cooperative types through an empirical exploratory study applying a random sampling survey. The results indicate that both contractual and relational governance have significant positive effects on member commitment, but vary with cooperative types. Specifically, there is a greater effect of contractual governance in company-affiliated cooperatives than in primary cooperatives, while the effects of relational governance increase in the order of company-affiliated, primary, and company-led cooperatives. Moreover, relational governance displays a greater positive influence on member commitment than contractual governance. These findings suggest that cooperatives should take organizational features, contractual and relational governance into consideration to improve member commitment and sustainable development.

## 1. Introduction

Farmers’ cooperatives (hereafter referred to as ‘cooperatives’) have substantially contributed to achieving the United Nations’s Sustainable Development Goals [1–3]. Reducing poverty and promoting food quality, members’ well-being as well as sustainable farm practices are some of the noticeable achievements of cooperatives [4–6]. Since cooperatives largely rely on members’ collective actions to achieve their organizational goals, their success and survival require high member organizational commitment [7]. Cooperatives with weak member commitment are more likely to have internal conflict and opportunistic behaviors.

Currently, cooperatives face the great challenge of declining member commitment due to increasing heterogeneity in members’ attitudes and perceptions resulting from their development and expansion [8]. This challenge is particularly great in China and other Eastern countries, as well as in the African region. In these areas, smallholders with high risk aversion and a lack of money and knowledge dominate the agricultural production sector [9]. Unlike user-owned and user-controlled cooperatives in Western countries, cooperatives are more like buyer-supplier alliances, with a few core members acting as initiators, owners, and controllers, as well as the majority of members serving only as users/ patrons [10, 11]. Additionally, since all types of agricultural operating subjects have been allowed to join cooperatives, cooperatives in China have become increasingly diverse and complex, with the appearance of many secondary cooperatives (i.e., cooperatives derived from the addition of other operating subjects on the basis of primary cooperatives (“cooperatives + farmers”) established by a horizontal union of farmers, such as “companies + cooperatives + farmers”, hereafter denoted by C+C+F). These cooperatives had a comparatively high level of member heterogeneity at the start of their existence. Consequently, cooperative firms are more likely to have incompatible interests with their members in these areas, posing a severe risk to member commitment to cooperatives [12].

Over the past few decades, an increasing number of studies have been conducted to investigate the determinants of member commitment. However, many studies addressing this topic have been theoretical [13] and carried out in Western countries such as the United States [14], France [15], and the Netherlands [16]. The way to govern the cooperative-member relationship has been convincingly shown to have significant impacts on member commitment [17]. For example, member commitment is positively influenced by their participation in cooperative governance [14, 16]. Surprisingly, while it is increasingly understood that governance mechanisms are critical to member commitment and cooperative performance [18, 19], very few studies have explicitly focused on how various governance mechanisms differentially affect member commitment.

The governance mechanisms of cooperatives can be divided into contractual and relational governance based on alliance governance theory. Contractual and relational governance have been extensively confirmed as critical predictors of collaboration outcomes such as collaboration satisfaction and relationship performance in interfirm alliances [20], but their relative effects on members’ continuance versus affective commitment have received scant attention. Moreover, whether they have a positive or negative effect on collaboration outcomes is highly controversial due to differences in things like specific transaction situations and theoretical model applications [20–23]. The organizational form of cooperatives is the vehicle for their transactions with members, and the various types of cooperatives differ in their underlying transactional features and context [24], which may affect the effectiveness of governance mechanisms. This raises the question: how do contractual and relational governance differentially affect member commitment in various types of cooperatives?

The objective of this study is therefore to investigate the relative impact of contractual and relational governance on members’ continuance and affective commitment in China’s cooperative context, as well as the moderating role of cooperative types in these effects. This study makes important contributions to the literature. First, many studies have addressed either member commitment or governance mechanisms but paid little attention to the governance mechanism-member commitment linkage. This study fills this gap by comparing the relative effects of contractual and relational governance on both members’ continuance commitment and affective commitment based on social exchange theory. Second, this study extends cooperative governance research and the application of alliance governance theory by analyzing cooperative governance mechanisms from the perspective of alliance governance. Prior research mainly investigated cooperative governance from the perspective of corporate governance, which highlights the owner and manager rather than the patron roles of members in cooperatives [25, 26]. Little work has focused on contractual and relational governance in cooperatives or individual-to-firm relationships. Third, it also contributes to the cooperative literature by innovatively dividing cooperatives into primary, company-affiliated, and company-led cooperatives and further revealing organizational types as key boundary conditions of the effect of governance mechanisms on member commitment.

## 2. Theory and hypotheses

### 2.1 Social exchange theory

Social exchange theory has been widely used to explain the effectiveness of contractual and relational governance [20]. The theory suggests that people become involved in and maintain an exchange relationship in the expectation of economic and social rewards [27]. Reciprocity is emphasized as the basic rule in exchange processes [28], and exchangers maintain a balance between giving and receiving [29]. Organizational commitment is regarded as a positive outcome [30]. When members are satisfied with the cooperative relationship, they are likely to show greater commitment in return for the cooperative’s friendly behavior [13]. In light of social exchange theory, cooperatives have exchange relationships with their members, and their governance behavior may influence member commitment by affecting the economic and social benefits that members receive from cooperatives. Thus, we draw upon social exchange theory to examine the relationship between governance mechanisms and member commitment in cooperatives.

### 2.2 Contractual governance and member commitment

Organizational commitment highlights the degree to which one partner is dedicated to a close and enduring relationship with another in cooperative relationships [31]. It has three dimensions: continuance, affective, and normative, respectively, indicating the extent to which members commit to cooperation as a result of economic benefits, emotional attachment, and responsibility norms [32]. Normative commitment is so hard to separate from affective commitment empirically that it has been dropped in empirical studies [33]. We thus focus only on members’ continuance and affective commitment toward cooperatives. Members’ continuance commitment refers to their preference to remain in cooperatives, as leaving would entail costs and the loss of acquired advantages, while their affective commitment emphasizes their wish to retain membership because of emotional attachment and belonging [15].

Contractual governance, a cooperation relationship governed by a contract [34], may have positive or negative effects on organizational commitment. It may restrain opportunism and safeguard cooperation by explicitly stating each party’s roles, responsibilities, punishments, and dispute resolution processes, but its rigid application may lead to trust deterioration, conflicts, and degraded cooperation [20, 35]. It has been found that contracts with control mechanisms can foster a client firm’s operational performance and satisfaction with its vendors in logistics outsourcing relationships [36], but contractual control increases conflict, resulting in decreased cooperation outcomes in R&D alliances [37]. According to social exchange theory, organizational commitment is closely related to cooperation performance, which in turn depends on the effectiveness of contractual governance. Therefore, contractual governance has ambiguous impacts on organizational commitment. When its positive impact dominates cooperation performance, it has positive effects on organizational commitment; otherwise, it has negative effects.

In the context of cooperatives, contractual governance is expected to foster member commitment by improving members’ cooperation satisfaction and performance. First, contractual governance in cooperatives is generally flexible. Agricultural contracts are characterized by significant incompleteness and high enforcement costs because it is challenging to monitor agricultural production and standardize agricultural products. As a result, cooperatives rarely establish rigorous and detailed contracts with members and strictly adhere to them. Second, due to their familiarity with other members and cooperatives rooted in the local community [2], members are likely to understand the original purpose of cooperative contractual governance and are less likely to regard contractual governance as a signal of mistrust, leading to conflicts and degrading cooperation. Therefore, cooperative contractual governance is more likely to play a positive role in cooperation performance as its flexible use may foster communication and cooperation confidence rather than increase opportunism and conflict by conveying distrust. Members tend to have higher affective and continuance commitment when they are satisfied with the cooperative and experience good profitability from cooperation [14, 38]. Contractual governance may thus positively affect member commitment. Therefore, we hypothesize the following:

H1: Cooperative contractual governance is positively associated with members’ continuance commitment (H1a) and affective commitment (H1b).

### 2.3 Relational governance and member commitment

Relational governance primarily uses relational norms or expectations to govern the behavior of exchange partners [39]. Its dimensions vary across research contexts because it is a multi-dimensional social-relation-based activity with strong situational dependence. For example, Poppo and Zenger [35] view relational governance as a composite factor with trust, open communication and sharing of information, dependence, and cooperation in an information-service exchange partnership, whereas Ju and Gao [40] regard it as a second-order factor with flexibility, information exchange, and solidarity in the exporter-distributor relationship. Overall, trust and communication (information exchange) are the most frequently used dimensions [20, 35]. In China’s cooperatives, trust and communication are vital dimensions of relational governance, as there is mutual trust and frequent communication among members who are primarily from the same village or town and have a high degree of kinship. Reputation is also an essential dimension, as members or managers in cooperatives face social sanctions such as gossip and exclusion if they betray relational norms [41]. Thus, we view relational governance as a composite factor involving trust, communication, and reputation in China’s cooperatives.

The impact of relational governance on organizational commitment is ambiguous. Relational governance has coordination and incentive functions [42] to improve cooperation satisfaction and performance [36], which are likely to promote organizational commitment. However, owing to the limitations of threats due to its ambiguous nature and easy abuse by opportunism [20], relational governance may diminish relationship performance [40] and may not or may even negatively affect organizational commitment. Wan et al. [42] found that coordinated relational governance strengthens the positive impact of material-specific investment on cooperation risks because key production information from one partner may be leaked and thereby abused opportunistically by the other. Hence, the impact of relational governance on organizational commitment depends on the net effect of its positive and negative roles.

We argue that relational governance has greater positive than negative impacts on member commitment in cooperatives. First, due to the effective role of social sanctions in cooperatives embedded in local communities, relational norms are effective in curbing opportunism [41]. Second, members’ information sharing is less likely to contribute to opportunism, as cooperatives have more information and technology advantages than members. Third, research has provided support for the positive influence of certain aspects of relational governance on member commitment. For instance, a theoretical study revealed that the cooperative’s reputation strengthens members’ continuance commitment [43]. Trust has a critical role in increasing the farmers’ willingness to cooperate [44], which contributes to farmer-members’ satisfaction and thereby enhances their long-term commitment [14].

Compared to contractual governance, relational governance is more likely to effectively increase member commitment in cooperatives. This is because cooperatives have social characteristics of community embeddedness [45], and relational governance has an advantage in managing cooperation relationships built upon a relational foundation. This may be particularly evident in China’s cooperatives that are embedded in local villages with a high level of Guanxi culture (i.e., a relational society) [10]. In contrast, contractual governance faces limitations in its ability to play a full role in cooperatives due to the difficulty and expense of formulating and implementing relatively appropriate agricultural cooperation contracts, as a result of the complexity of agricultural production, the low legal literacy of farmers, and an imperfect legal and regulatory system. Therefore, we propose the following hypothesis:

H2: Cooperative relational governance is positively associated with members’ continuance commitment (H2a) and affective commitment (H2b).
H3: Relational governance more effectively improves both members’ continuance commitment (H3a) and affective commitment (H3b) than contractual governance in cooperatives.

### 2.4 The moderating role of cooperative types

China has witnessed a boom in cooperatives since the Cooperative Law took effect in 2007. Cooperative organization forms have evolved from the early pattern of mainly primary cooperatives to a situation where primary and secondary cooperatives coexist. Some primary cooperatives cooperate with or have even set up their own agricultural companies to improve their operational strength or market recognition. Some companies cooperate with cooperatives to overcome the shortcomings of the “company + farmers” alliance with high transaction costs and default rates. The number of secondary cooperatives involving the C+C+F pattern has increased as it integrates the operation advantages of household, cooperation, and company [46]. However, both primary and secondary cooperatives are at relatively low levels of development [47], with cooperation problems such as high default rates and low product delivery.

Cooperatives are typically distinguished by the dominant position of a few core members in China, such as common-farmer-led, rural-elite-led, enterprise-led, and related-organization-led cooperatives [47]. However, agricultural operation subjects vary in cooperation strengths and objectives [46], and their participation may affect cooperative operational characteristics even without them being leading subjects. Thus, the distinctions in cooperatives should take member type into consideration. China’s agricultural operational subjects primarily encompass farmers, cooperatives, and agricultural enterprises. Farmers are the basic subject of cooperatives due to their unique contracting rights over rural land. Enterprises are important participants in cooperatives because they can improve the operational strength or market recognition of cooperatives and obtain stable supplies of products of both quantity and quality. As primary cooperatives and secondary cooperatives of the C+C+F pattern are the main forms of China’s cooperatives, our study focuses on these cooperatives, further distinguishing them into three types based on their leading subjects and member types. They are primary cooperatives, company-affiliated cooperatives (secondary C+C+F cooperatives dominated by cooperatives), and company-led cooperatives (secondary C+C+F cooperatives dominated by companies).

Cooperative types are expected to moderate the relationship between governance mechanisms and member commitment in cooperatives. According to social cognitive theory, the environment shapes human cognition [48]. Since member commitment is a member’s cognitive attitude toward cooperatives, and cooperative types imply different institutional environments, cooperative types may differentially affect members’ cognition toward their governance mechanisms and thereby influence the governance mechanism-member commitment linkage. Specifically, primary cooperatives are typically composed of farm members who share high similarities in their needs and interests. They provide primarily basic production services, such as agricultural input supply and technology exchange, with simple internal transaction relationships. As a result, they are more likely to create a good atmosphere of cooperation, and internal cooperation conflicts and risks are usually low. In contrast, secondary cooperatives with company participation usually have a larger business content and scope of interests. They often have higher asset specificity, greater member heterogeneity, and more complex interests. Consequently, they are more susceptible to cooperative risks and conflicts compared to primary cooperatives [24, 42]. This is more noticeable in company-led cooperatives than in company-affiliated cooperatives. Company-affiliated cooperatives are led by cooperatives and usually engage in the simple processing and distribution of agricultural products. Company-led cooperatives are led by companies, which aim to ensure a stable supply of raw materials and maximize value addition throughout the entire industry chain. As a result, company-led cooperatives typically require more specific investments in technology, equipment, and so on. In addition, in company-led cooperatives, the actual controllers of companies are generally back-to-home or even non-local entrepreneurs, resulting in weaker relationships with members. Therefore, the cooperation risks of company-affiliated cooperatives tend to be higher than those of primary cooperatives but lower than those of company-led cooperatives. The higher the risks of cooperation, the greater the organization’s need for and sensitivity to governance mechanisms [36, 49]. Consequently, the use of governance mechanisms becomes more pressing and impactful in such situations. It can be inferred that the positive impact of governance mechanisms on member commitment is likely to be weaker for company-affiliated cooperatives than for company-led cooperatives, but it may be stronger for company-affiliated cooperatives than for primary cooperatives. Consequently, we propose the following hypotheses:

H4: Cooperative types moderate the relationship between contractual governance (H4a) / relational governance (H4b) and member commitment.
H5: The positive relationship between contractual governance and members’ continuance commitment (H5a)/ affective commitment (H5b) in company-affiliated cooperatives is stronger than in primary cooperatives, but smaller than in company-led cooperatives.
H6: The positive relationship between relational governance and members’ continuance commitment (H6a)/ affective commitment (H6b) in company-affiliated cooperatives is stronger than in primary cooperatives, but smaller than in company-led cooperatives.

## 3. Methodology

### 3.1 Participants and procedures

The data were collected from a face-to-face survey of China’s cooperatives. Given the differences between east, center, and west China as well as the feasibility of a field survey, we randomly selected seven prefecture-level cities in the provinces of Guangdong, Jiangxi, Guangxi, and Guizhou to serve as survey regions. To ensure the reliability and validity of the survey data, we randomly selected cooperatives from a list of cooperatives obtained from government websites in these survey areas. We obtained the contact information of the selected cooperatives and invited them to participate in our face-to-face investigation with the help of relevant government departments. To lessen respondents’ guard and concealment mindset, we explicitly explain the purpose of the research and the confidentiality of the information to respondents and collect information in a relaxed and friendly chat-style questionnaire interview approach. The procedure of data collection followed all ethical guidelines and standards of the research and obtained ethical approval from the ethics committee of South China Agricultural University. Verbally informed consent was obtained from all respondents involved in our study.

The target respondents were cooperative chairpersons, chosen for their comprehensive understanding of organizational characteristics, governance mechanisms, and member commitment because they are frequently not only the direct managers of members but also their friends, neighbors, or relatives. It is notable that organizational commitment can be measured using both self-rated methods [32] and other-evaluation methods [50], and that self-rated commitment and managerial perceptions of member commitment are significantly correlated [50]. This is because organizational commitment can easily be reflected by revealed behavior [51]. Given that member commitment is the only variable directly relating to members in this study, we adopted other-evaluation methods and asked chairpersons to evaluate the overall member commitment toward cooperatives. Ultimately, we obtained a database comprising 232 cooperatives.

### 3.2 Measures

Member commitment, as stated above, was measured using the other-evaluation method. Members’ continuance and affective commitment were scored on 5-point Likert scales consisting of four items and three items, respectively. These scales were adapted based on the scales of Allen and Meyer [32] and the characteristics of China’s cooperatives. To align with the other-evaluation method, the subject of the items in scales was changed to farmer members following Yun et al. [50]. An example item from the continuance commitment scale reads as follows: “Member income will decrease if they quit the cooperative”.

Relational governance is a second-order construct composed of three dimensions: trust, communication, and reputation, as mentioned earlier. In accordance with Lee and Kim [52], Ganesan [53], as well as Ju and Gao [40], combined with the characteristics of cooperative operation and governance, a 5-point Likert scale consisting of 13 items was developed. A sample item reads, “Our farmer members are sincere and trustworthy.”

Contractual governance is divided into quality control intensity and residual settlement incentives because contracts in cooperative-member relationships are usually rough or even verbal, and their governance role depends on core provisions that are closely related to their cooperation costs and benefits. As explained by Liang et al. [54], given that farmer members care most about the actual payments, governance that allocates a higher share of profits to members can incentivize them to make more commitments.

Four sub-indicators were established to measure the intensity of quality control according to the content of quality control: the purchase and use requirements of agricultural input, the intensification level of quality control ability and willingness. The first two were measured as the average score of the purchase and use requirements of fertilizer/feed and pesticide/epidemic control drugs, respectively (no requirement = 1; simple requirement = 2; specified higher high standards = 3; uniform supply/use = 4; pursuit of high quality without use = 5). The latter two were measured as the sum of the scores of three and four binarily variables, respectively: whether the cooperative has established production procedures, conducted technical training, and provided production guidance; and whether the cooperative has adopted production supervision, violation punishment, quality inspection, and quality incentive measures.

Three sub-indicators were established to measure residual settlement incentives based on the interest linkage between cooperatives and members: production service and product transaction settlement incentives, as well as earnings distribution incentives. Specifically, the production service settlement incentive was measured as the sum of the scores of four binary variables that ask whether the cooperative provides preferential procurement services for seedlings/breeding poultry, fertilizers/feed, and epidemic control drugs, as well as preferential agricultural machinery services. The product transaction settlement incentive was measured based on the form of the purchasing price (no acquisition = 0; market price = 1; guaranteed price = 2; fixed price/market price + additional price = 3). The earnings distribution incentive was measured by the distribution form (no distribution = 0; only by capital share = 1; mainly by capital share = 2; mainly by trading volume = 3). Finally, we used an entropy weight method to sum the seven sub-indicators to measure contractual governance, with the maximum possible value of 4.52.

Cooperative types were identified based on the relationship between the cooperative and the company and measured with a dummy variable. If cooperatives have no long-term cooperation with companies, they belong to primary cooperatives and are defined as 0. If a company was established by the cooperative, then it was designated a company-affiliated cooperative and was defined as 1. If the cooperative was established by the company or has a contractual cooperation relationship with the company, it is designated a company-led cooperative and is defined as 2.

As organizational characteristics may affect the output and results of cooperative behaviors [54], we included control variables such as the age of cooperatives, membership size, chairperson’s capital shares, and demonstration level of cooperatives. We defined a dummy variable to indicate the demonstration level of cooperatives (0 = non-demonstration, 1 = prefecture/district level, 2 = provincial/national level). Other control variables are continuous variables, and their actual values are used to measure them.

## 4. Results

### 4.1 The common method bias test

Given that the collected data were single-sourced and self-reported, we conducted a Harman’s single-factor test to assess potential common method biases [55]. We carried out an exploratory factor analysis without rotation using all the measurement items of the constructs. The result revealed that the first factor explained only 35.59% of the variance, which is less than the recommended value of 50%. Therefore, this test indicated that there is no serious common method bias problem in this study.

### 4.2 Reliability and validity analyses

Cronbach’s α and composite reliability (CR) were employed to examine reliability. The results presented in Table 1 show that all Cronbach’s α values were above 0.6, and the CR value ranged from 0.791 to 0.900, indicating sound reliability across all variables. Moreover, the square root of the average extraction variance of each variable was greater than the correlation coefficient between the variable and other variables (see Table 1), which indicates that each variable has good discriminant validity.

**Table 1:** Results of reliability and validity analysis.

Variable	Cronbach’s α	CR	AC	CC	TR	CO	RE
AC	0.821	0.900	*0*.*866*				
CC	0.640	0.791	0.461	*0*.*699*			
TR	0.785	0.856	0.392	0.625	*0*.*737*		
CO	0.736	0.837	0.222	0.505	0.405	*0*.*751*	
RE	0.814	0.880	0.405	0.607	0.564	0.503	*0*.*804*

**Note:** CR = composite reliability; AC = affective commitment; CC = continuance commitment; TR = trust; CO = communication; RE = reputation. The square roots of the average extraction variance are shown in italics on the diagonal.

### 4.3 Descriptive statistics

Descriptive statistics for all variables are presented in Table 2. The age of cooperatives ranged from 1 to 13, consistent with the sample cooperatives of Liang et al. [54]. The average membership size was 125.47. The chairperson’s capital shares ranged from 0 to 100%, with an average of 51.06%, indicating that chairpersons held the majority of the cooperative capital shares. The average values of contractual and relational governance were 1.99 and 4.28, respectively, implying that China’s cooperatives lean more toward relational governance than contractual governance. The average values of continuance and affective commitment were 3.47 and 4.13, respectively, suggesting that members’ continuance commitment is generally lower than their affective commitment.

**Table 2:** Descriptive statistics of variables.

Variable	Mean	Standard deviation	Min.	Max.
Age of cooperatives	7.33	2.79	1	13
Membership size	125.47	107.63	5	600
Chairperson’s capital shares (%)	51.06	33.33	0	100
Demonstration level	1.54	0.68	0	2
Cooperative type	0.90	0.83	0	2
Contractual governance	1.99	0.60	0.24	3.24
Relational governance	4.28	0.44	2.69	5
Affective commitment	4.13	0.75	1.33	5
Continuance commitment	3.47	0.79	1.25	5

### 4.4 Hypotheses testing

Ordinary least squares estimation was used to test the hypotheses, and the regression results are presented in Table 3. We examined possible multicollinearity among the variables and estimated the coefficients with robust standard errors. The results show that there are no multicollinearity problems, as each variance inflation factor is far below 10. All of the regression models have significant F-statistic values at the 1% level, indicating a good overall fit.

**Table 3:** Regression results.

Variable	Continuance commitment				Affective commitment			
	Model 1	Model 2	Model 3	Model 4	Model 5	Model 6	Model 7	Model 8
	FS	PC	CAC	CLC	FS	PC	CAC	CLC
Contractual governance	0.319*** (3.89)	0.347*** (2.67)	0.394*** (2.71)	0.066 (0.35)	0.170*** (2.81)	0.184*** (2.27)	0.257*** (2.18)	0.042 (0.32)
Relational governance	0.688*** (6.32)	0.700*** (3.62)	0.597*** (3.11)	0.856*** (4.41)	1.193*** (15.00)	1.085*** (8.98)	1.011*** (6.50)	1.495 (11.07)
Age of cooperatives	−0.014 (−0.74)	−0.002 (−0.07)	−0.040 (−1.13)	0.010 (0.28)	−0.018 (−1.24)	−0.004 (−0.21)	−0.075** (−2.60)	0.025 (0.94)
Membership size	0.001 (1.12)	0.001 (0.81)	0.000 (0.31)	0.001 (0.77)	0.001*** (4.66)	0.001*** (3.04)	0.001** (2.36)	0.001 (2.09)
Chairperson’s capital shares	0.000 (0.28)	0.002 (0.95)	0.002 (0.68)	−0.004 (−1.42)	0.000 (0.27)	0.001 (0.48)	−0.001 (−0.51)	−0.001 (−0.45)
Demonstration level	−0.068 (−0.87)	−0.116 (−0.90)	−0.001 (−0.01)	−0.041 (−0.25)	0.035 (0.52)	−0.042 (−0.52)	0.305** (2.58)	−0.076 (−0.65)
Cooperative type = CAC	−0.171 (−1.60)				−0.202*** (−2.45)			
Cooperative type = CLC	−0.214* (−1.78)				−0.224*** (−2.63)			
Constant	0.203 (0.40)	0.024 (0.03)	0.258 (0.25)	−0.213 (−0.22)	−1.318*** (−3.60)	−0.837 (−1.56)	−1.150 (−1.40)	−2.508 (−3.66)
*R* ^2^	0.248	0.266	0.276	0.274	0.575	0.584	0.527	0.682
*F*-value	10.240***	5.190***	4.000***	3.900***	35.540***	20.080***	11.700***	22.150***

**Note:** FS = full sample; PC = primary cooperatives; CAC = company-affiliated cooperatives; CLC = company-led cooperatives; The benchmark category for cooperative types is primary cooperatives.

*, **, and *** represent the significance levels of 1%, 5%, and 10%, respectively; the robust standard errors are in parentheses.

As Model 1 in Table 3 shows, the coefficients of both contractual and relational governance are positive and significant (β = 0.319, p<0.001; β = 0.688, p<0.001), suggesting that both contractual and relational governance have significant positive impacts on members’ continuance commitment. Thus, hypotheses 1a and 2a were supported. Model 5 in Table 3 shows that both contractual and relational governance are positively related to members’ affective commitment (β = 0.184, p<0.001; β = 1.085, p<0.001), supporting hypotheses 2a and 2b. Furthermore, the regression coefficient of relational governance is greater than that of contractual governance in both Model 1 and Model 2. This indicates that the positive effect of relational governance on members’ continuance and affective commitment is stronger than that of contractual governance. Therefore, hypotheses 3a and 3b were verified. The control variables of cooperative type and membership size both significantly influence member commitment. Members’ affective commitment is higher in primary cooperatives than in company-affiliated and company-led cooperatives, and members’ continuance commitment is lower in company-led cooperatives than in primary cooperatives. The membership size is positively related to members’ affective commitment.

As cooperative type is a categorical variable, we examined its moderating effect in accordance with Cohen and Cohen [56]. Grouping regression analysis was first performed according to cooperative types, as shown in Models 2–4 and Models 6–8 of Table 3. The Z-test was then used to examine the significance of the variations in regression coefficients in different models (see Table 4). As Table 3 shows, the regression coefficients of contractual governance are positive and significant in Models 2–3 and 6–7 but not in Models 4 and 8, and the regression coefficients of contractual governance are greater in Models 3 and 7 (β = 0.394, p<0.001; β = 0.257, p<0.001) than in Models 2 and 6, respectively (β = 0.347, p<0.001; β = 0.184, p<0.001). The Z-values of the variations in regression coefficients of contractual governance between any two models in Model 2–4 or Model 6–8 exceed 1.96 (see Table 4), suggesting that there are significant differences in the impact coefficient of contractual governance on both continuance and affective commitment in these models. These results indicate that cooperative types play a significant moderating role in the positive impact of contractual governance on members’ continuance/ affective commitment, with a greater positive effect for company-affiliated cooperatives than for primary cooperatives. Thus, hypothesis 4a was verified, and hypothesis 5 was rejected. As seen in Table 3, the regression coefficients of relational governance are positive and significant for Models 2–8, and the regression coefficients of relational governance for Model 2 and Model 6 (β = 0.7000, p<0.001; β = 1.085, p<0.001) are larger than those for Models 3 and Model 7 (β = 0.597, p<0.001; β = 1.011, p<0.001), respectively, and smaller than those for Models 4 and Model 8 (β = 0.856, p<0.001; β = 1.495, p<0.001), respectively. The Z-value of regression coefficient variations of relational governance in any two models of Models 2–4 or Models 6–8 surpass 2.56 (see Table 4), indicating that the regression coefficients of relational governance differ significantly in Models 2-4/Models 6–8. These results reveal that cooperative types significantly moderate the relationship between relational governance and members’ continuance/affective commitment; their positive effects increase in that order for company-affiliated, primary, and company-led cooperatives. Therefore, hypothesis 4b was supported and hypothesis 6 was not verified.

**Table 4:** The Z-values of the regression coefficient variations.

Explanatory variable	Members’ continuance commitment			Members’ affective commitment		
	*Z*_2-3_	*Z*_2-4_	*Z*_3-4_	*Z*_6-7_	*Z*_6-8_	*Z*_7-8_
Contractual governance	2.136	10.750	11.569	4.458	8.027	10.250
Relational governance	3.368	5.083	7.910	3.304	19.957	19.584

**Note**: *Z*_a-b_ represents the Z value of the regression coefficient variation in the explanatory variables in Model a and Model b of Table 3, and *a* and *b* fall in the intervals [2, 4] and [6, 8].

## 5. Discussion and implications

### 5.1 Discussion of findings

First, relational governance has a significant positive impact on member commitment. As relational governance improves, so do the members’ continuance and affective commitment. Cooperatives are characterized by extensive inner social ties; their inner mutual trust and communication contribute to fostering member loyalty and thus member commitment [2]. Furthermore, social control manifested through reputation encourages collective action by increasing the deterrent cost associated with free riding and defaults. Thus, relational governance plays a great role in cooperative governance. This finding supports previous research that emphasizes the significant role of social relationships in China’s cooperatives, as well as the positive outcomes that can be achieved through social capital, such as increased member participation in collective activities and improved cooperative economic performance [41].

Second, contractual governance also has a significant positive effect on members’ continuance and affective commitment, but its effect is weaker than that of relational governance. This could be due to the members’ economic activities being embedded in the social context of the cooperative’s community [2]. Contractual governance has a mandatory characteristic and relies on the binding force of authority to govern transaction relationships [35]. This is useful for curbing opportunistic behaviors and so improving cooperation performance, but it may not match very well with cooperatives’ community-based member relationships, particularly in China’s cooperatives with the Guanxi style of interpersonal communication. Contractual governance primarily fosters member commitment by offering better economic benefits and generating greater satisfaction. Similar studies have also found that relational governance generates better cooperation satisfaction than contractual governance in China’s logistics outsourcing alliance [36]. In spite of this, it is crucial to reinforce the positive effect of contractual governance on member commitment in consideration of long-term development. This is necessary because relational governance has flaws like rising marginal costs and insufficient binding force, and its influence on member commitment may gradually decrease as membership size grows.

Third, cooperative types moderate the relationship between governance mechanisms and member commitment. The contractual governance of company-affiliated cooperatives has stronger positive effects on member commitment than does that of primary cooperatives. The positive effect of relational governance on member commitment in primary cooperatives is stronger than in company-affiliated cooperatives but weaker than in company-led cooperatives. In company-led cooperatives, contractual governance cannot significantly promote member commitment because it may signal distrust toward members and provoke conflicts, as most companies are not from local villages or towns and have relatively weak relationships with members. Company-affiliated cooperatives are less sensitive to relational governance than primary cooperatives because they mainly originated in primary cooperatives and have accumulated better internal relationships. Consequently, the positive impact of relational governance on member commitment in company-affiliated cooperatives is lower than in primary cooperatives. As Cao and Lumineau [20] argued, it is vital to perform a scientific combination of contractual and relational governance depending on contextual circumstances since they differ in impact mechanisms, limitations, and adaptability in promoting cooperative relationships.

### 5.2 Theoretical implications

This study has several significant theoretical implications. First, this study extends the organizational commitment literature and the application of social exchange theory by empirically revealing the linkage between governance mechanisms and organizational commitment in the context of cooperatives. While previous studies have extensively explored the influencing factors of organizational commitment using social exchange theory [57], most of them have focused on investor-owned firms and overlooked the role of governance mechanisms in shaping organizational commitment. The unique characteristic of cooperatives lies in the fact that their members are the main owners, managers, and patrons [13]. This uniqueness makes member commitment more critical and the formation mechanism more complex. Moreover, as an important means to coordinate the relationship between subjects, governance mechanisms significantly impact the behavior, attitude, and cooperation performance of the involved parties [20, 58]. This study applied social exchange theory to investigate how governance mechanisms affect member commitment to cooperatives. The results highlight the pivotal role of governance mechanisms as influential factors in shaping the exchange relationship between cooperatives and their members, ultimately impacting member commitment. This not only contributes to our understanding of the antecedents of organizational commitment in the context of cooperatives but also provides new insights into social exchange theory.

Second, this study contributes to the cooperatives’ governance literature and alliance governance theory by analyzing cooperative governance from the perspective of alliance governance and comparing the relative effects of contractual and relational governance on members’ continuance and affective commitment. Previous research has recognized the crucial role of governance in cooperatives, but most studies have focused on governance structure [47, 59] or deconstructed governance mechanisms into variables such as ownership structure and council structure based on corporate governance theory [5]. As noted earlier, the cooperative-member relationship in Eastern countries resembles more of a buyer-supplier relationship than an ownership relationship [60]. The formal rules regarding governance structure almost exist in name only. It is more appropriate to divide their governance mechanisms into contractual and relational governance, based on alliance governance theory. In addition, prior studies have mostly investigated the impacts of contractual and relational governance on collaboration outcomes in the context of various interfirm alliances, and the results are controversial due to differences in theoretical models and research contexts [20]. Given this fact, we investigate the effect of both contractual and relational governance on member commitment from an alliance governance perspective. This offers a new perspective to explore cooperatives’ governance and contributes to the generalizability of contractual and relational governance in a new organizational context.

Third, this study highlights the importance of cooperative context by identifying organizational types as a key boundary condition for the impact of governance mechanisms on member commitment. Given the fact that China’s cooperatives are obviously different from cooperatives in Western countries and present a diversified development trend, many scholars have called for attention to the heterogeneity of cooperatives and their indigenous research [41, 61]. We answer these calls by innovatively categorizing cooperative types as primary, company-affiliated, and company-led cooperatives based on China’s cooperative characteristics and revealing its moderating influence on the relationship between governance mechanisms and member commitment. This contributes to a deeper understanding of China’s cooperatives and enriches cooperative theory.

### 5.3 Managerial implications

The results offer some valuable managerial insights. First, China’s cooperatives should attach importance to the use of relational governance to strengthen member commitment. In all kinds of cooperatives analyzed in the study, the promotion impact of relational governance on both members’ continuance and affective commitment is significantly greater than that of contractual governance. Therefore, managers should focus on actively enhancing communication and trust between cooperatives and members, vigorously building a good social reputation for serving members, and creating a good cooperative atmosphere to improve member commitment. Moreover, cooperatives need to focus on improving the usability and effectiveness of contractual governance. As cooperatives develop and member size expands, the relational governance characteristics of increasing marginal cost and insufficient binding force may gradually weaken their governance effect. Therefore, cooperatives need to gradually strengthen the promotion effect of contractual governance on member commitment in consideration of long-term development. Finally, cooperatives should perfect their governance mechanisms according to their organizational types. Blindly pursuing a high-intensity governance mechanism may not be beneficial, particularly for company-led cooperatives, where contractual governance may not significantly improve both members’ continuance and affective commitment. Therefore, managers should tailor their governance approach based on their cooperative’s organizational type.

### 5.4 Limitations and future research directions

Despite yielding meaningful findings, this study has some limitations that are worth further exploration in the future. First, a static model and cross-sectional data were used in this study, which helps boost generalizability but is unable to reflect the dynamic relationships between cooperatives and members. In the future, longitudinal data can be collected to explore the dynamic relationship between governance mechanisms and member commitment. Second, the study only investigated the influence of organizational types on the relationship between governance mechanisms and member commitment. Future research could further explore the moderating role of other environmental factors, such as organizational culture and environmental dynamics. Third, the internal relationship between contractual and relational governance was ignored. Further studies can explore the substitution or complementary relationship between the two governance mechanisms in terms of their impact on member commitment in different life cycle stages or institutional environments of cooperatives.

## 6. Conclusions

Cooperatives are becoming increasingly popular worldwide due to their advantages in addressing sustainability concerns such as economic inequality and poverty. As cooperatives are owned and patronized by their members, member commitment is essential for their development. As such, this study developed and tested a model based on social exchange theory to explain how contractual and relational governance differentially affect member commitment. The results reveal that both contractual and relational governance have significant positive effects on member commitment, and relational governance is more effective in promoting both members’ continuance and affective commitment compared to contractual governance. In addition, these effects are moderated by organizational types. Specifically, China’s cooperatives are categorized as primary, company-affiliated, and company-led cooperatives based on their leading subjects and member types. Contractual governance has a stronger positive impact on member commitment in company-affiliated cooperatives than in primary cooperatives. The positive impacts of relational governance on member commitment improve in the order of company-affiliated, primary, and company-led cooperatives. These findings highlight the relatively different effects of contractual and relational governance on member commitment, which also vary with cooperative types. It is critical to match appropriate contractual and relational governance according to their organizational features to attain better member commitment and sustainable development in cooperatives.

## Supporting information

S1 File (ZIP)
